# 

**Published:** 1871-05

**Authors:** 


### THE AMERICAN JOURNAL OF DENTAL SCIENCE,

EDITED BY

F. J. S. GORGAS, M. D., D. D. S.

VOL. 5.—THIRD SERIES.—NO 12.

Surgeon Genl's Office LIBRARY 39615 Washington D.C.

BALTIMORE:
SNOWDEN & COWMAN.
LONDON:
TRUBNER & CO., 60 PATERNOSTER ROW.
1872.

31

3375-b

### INDEX TO VOL. V.--THIRD SERIES.

A Bullet makes Eight Wounds. - - - - - 284
A Cheap Retort. - - - - - - - - - 87
A Method of Taking Impressions. - - - - - 143
A New Anæsthetic. - - - - - - - - - 85
A New Filling. - - - - - - - - - 524
A New Styptic. - - - - - - - - - 236, 572
A Novel Surgical Operation. . . . . 571
A Remedy for Sea Sickness. - - - - - - - - 87
Abstinence Extraordinary. - - - - - - - - 89
Abuse of Alcohol among Women. - - - - - - 138
Abell. A. B., Jr., D.D.S. - - - - - - - - - 471
Address before the Virginia State Dental Association. - 391
Address. Extracts from, Dr. O. W. Holme's. - - - 542
Alabama Dental Association. - - - - - - 14, 463
Alloying and Refining Gold for Dental Purposes. - - 1
Alveolar Abscess. - - - - - - - - - 48, 289
Alveolar Abscess with Necrosis of the Maxilla. - - - 279
Amalgam Experiments. - - - - - - - - - 361
American Academy of Dental Science. - - 239, 307, 335
American Dental Association. 59, 112, 189, 264, 308, 343, 400
American Dental Convention. - - - - - - 62, 113
Anæsthetics. - - - - - - - - - - 377
Ancient Dentistry. - - - - - - - - - - 377
Andrews. E. H., M.D. - - - - - - - - 516
Antrum. Disease of the - - - - - - - - 216
Antrum of Highmore and its Diseases. - - - - - 506
Are Artificial Teeth Capable of Producing Salivation? - 173
Arsenic. Test for - - - - - - - - - - 427
Arthur's System of Treatment for the Prevention of Decay of Teeth. . . . . . . . . . 558
Articulating Artificial Teeth in the Mouth. - - - - 471
Austen. Prof. P. H., M.D., D.D.S. - - - - - - - 97
Baltimore College of Dental Surgery. - - - - - 497
Barker. Geo. T., D.D.S. - - - - - - - - - 217
Beers. W. G., L.D.S. - - - - - - - - - 179
Blake. Dr. E. - - - - - - - - - - - 205
Bleaching Beeswax. - - - - - - - - - 44
Bleaching Teeth. - - - - - - - - - - 42
Bliss. D. W., M.D. - - - - - - - - - 273
Bond. O. J., D.D.S. - - - - - - - - - 193
Bone Felon. - - - - - - - - - - - 330
Bone Fibrous. - - - - - - - - - - - 49
Boston Dental College. - - - - - - - - 62

Boyd. Dr. H. D. - - - - - - - - - - 216
Brown. M. J., D.D.S. - - - - - - - - - 1
Buckingham. T. L., D.D.S. - - - - - - - - 183
Bullet over Nineteen Years in the Brain. - - - - 428
Burkholder, N. M., D.D.S. - - - - - - - - 493
Burns and Scalds. - - - - - - - - - - 523
Burring Machine. - - - - - - - - - 114
BIBLIOGRAPHICAL.
Harris' Principles and Practice of Dentistry. - - 190
Insanity in Women. - - - - - - - 191
Diseases of the Womb - - - - - - - 191
Virginia Clinical Record. - - - - - - 191
Medical Cosmos. - - - - - - - - - 191
American Sournal of Microscopy. - - - - - 191
Medical World. - - - - - - - - - 191
Taking Impressions of the Mouth. - - - - - 239
Review of Darwins' Theory of Origin and Development of Man. - - - - - - - - - - 239
Plastics and Orthopedics. - - - - - - - - 239
New Remedies. - - - - - - - - - 239
Practical Therapeutics. - - - - - - - - 378
The Teeth and How to Save Them. - - - - 378
Treatment and Prevention of Decay of the Teeth. - 378
Transactions of Southern Dental Association, 1871. - 429
Physical Diagnosis of Brain Diseases. - - - - 429
Clinical Examination of Urine, - - - - - 429
Syphilitic Epilepsy. - - - - - - - - - 429
Sugar Formation in the Liver. - - - - - 429
Transactions of the American Opthalmological Society. 429
Treatment of Versions of Unimpregnated Uterus. - 429
American Agriculturist. - - - - - - - - 429
Prevention of Abscess in Hyperdermic Medication. 429
Mutual Relations of the Medical Profession. - - 430
Medical Education in America. - - - - - 430
Vick's Illustrated Catalogue and Floral Guide. - 430
Calcified Pulp. - - - - - - - - 45, 93
California Quicksilver. - - - - - - - - 41
Cancrum Oris. - - - - - - - - - - 44
Cases of Cancer Treated with Cundurango. - - - - 276
Case of Triple Fracture of Lower Jaw. - - - - - 15
Caries and Necrosis. - - - - - - - - 16
Carbolic Acid Preparations. - - - - - - - 40
Castor Oil. - - - - - - - - - - - 138
Caries. Dental, - - - - - - - - - - 438
Carbuncles. - - - - - - - - - - - 186
Carbon Bisulphide, Rhigolene, etc., as Local Anæsthetics. 523
Cause of the Degeneracy of Teeth, Especially among Children. 481
Caries and Removal of Whole of Inferior Maxilla. - - 273

Cell or Skin Grafting. . . . . . . 572
Charlatans and Empirics, or Dentistry as a Learned Profession. 143
Chloral. - - - - - - - - - - - 225
Chloral in Toothache. - - - - - - 334, 439, 571
Chloral as an Antiseptic. - - - - - - 423, 428
Chloral. Preparation of, - - - - - - - - - - 24
Chloride of Zinc with Chlorate of Potassium. - - - - 523
Chloroform. The Tolerance of, - - - - - - - - 137
Chuypein. Dr. T. F., - - - - - - - - - - - 61
Celluloid Base. - - - - - - - - - 224, 468, 519
Clark. F. Y., M.D., D.D.S. - - - - - - - - 250
Class Address. - - - - - - - - - - - - 529
Cleansing Agent. An Excellent, - - - - - - - 473
Cleft Palate. - - - - - - - - - - - - 8, 556
Cobweb in Hemorrhage from Extracting Teeth. - - 480
Cobb. S. J., D.D.S. - - - - - - - - - - 461
Concealed Vascular Tumor of the Face. - - - - - 188
Condurango. - - - - - - - - - - - - 188
Conservative Dentistry. - - - - - - - - - 51
Cotton to Stop Bleeding. - - - - - - - - 477
Cotton. Styptic, - - - - - - - - - - - 573
Creosote in Mercurial Salivation. - - - - - - 88
Croup. Treatment of, - - - - - - - - - 423
Cummings Patent. - - - - - - - - 478, 524, 574
Cushing. G. H., D.D.S. - - - - - - - - 51
Cutler. S. P. M.D., D.D.S. - 63, 105, 241, 337, 357, 361, 385, 481
Dangers and Fatal Results from Hydrate of Chloral. - 90
Davis. Dr. R. B. - - - - - - - - - - - - 300
Death from Alveolar Hemorrhage. . . . . . 576
Death from Chloroform. - - - - - - - - - 43
Death from Chloroform. One of the Causes of, - - - - 168
Death from Chloroform. Cause of, - - - - - - - 426
Death in a Dental Office. - - - - - - - - - 286
Death from Nitrous Oxide. - - - - - - - - 575
Death from Ether. - - - - - - - - - - - 44
Death from Methylene. - - - - - - - - - 477
Dental Caries. - - - - - - - - - - - - - 438
Dental Hygiene. - - - - - - - - - - - 70
Dental Pulp. Pathological Condition of the, - - - - 337
Dental Pulp. Treatment of, - - - - - - - - 130
Dental Therapeutics. - - - - - - - - - - - 250
Dentistry. - - - - - - - - - - - - - 97
Dentistry in the United States. - - - - - - - 376
Dentistry and its Difficulties. - - - - - - - - 431
Discolored Teeth. Treatment of, - - - - - - - 42
Diet Treatment of Decayed Teeth. - - - - - - 166
Disinfecting Cotton. - - - - - - - - - - - 283

Dental Items. - - 45, 46, 47, 48, 90, 93, 94, 139, 142, 143, 332, 333, 334, 335, 380, 381, 382, 384
Destroying Nerves. . . . . . 554
Eames. W. H., D.D.S. - - - - - - - 468, 513
Earache in Children. - - - - - - - - 474
Earth Eating. - - - - - - - - - 85
Eastern Ohio Dental Association. - - - 359, 465, 540
Effects of Alcohol and Tobacco on the Sight. - - 136, 234
Enormous Collection of Earthy Matter in a Human Lung. - 425
Epithelioma. The course of, - - - - - - - - 185
Epithelioma Removed by Bromine. - - - - - - 284
Epulis. - - - - - - - - - - - - - 472
Errata. - - - - - - - - - - - 528
Excision of the Inferior Maxillay Nerve. - - - - 31
Exposed Pulps. - - - - - - - - - - 433
Ether Spray in Ulcers and Cutaneous Affections. - - 88
Extracting Teeth During Pregnancy. - - - - - - 166
Extraction of Teeth—When is it Indicated? - - - - 499
Evans, Thos. W., M.D., D.D.S. - - - - - - 384
Facial Neuralgia Treated by Extirpation of Superior Maxillary Nerve. - - - - - - - - - - - - 414
Fatal Salivation from Bichloride of Mercury. - - - - 475
Ferguson. T. H., D.D.S. - - - - - - - - - 529
Fibro-Cystic Tumor of Inferior Maxilla. - - - - - 417
Fibrous Tumor. Removal of, from Roof of Mouth. - - 431
Filling Teeth. - - - - - - - - - - 452
Fogle. Wm. J. D.D.S. - - - - - - - - - 8
Foil Cylinders. . . . . . . . 574
Folsom's Improved Atmospheric Dental Plates. - - 513
Ford. Arthur C., D.D.S. - - - - - - - - 433, 534
Fracture of a Rib from Coughing. - - - - - - 138
Georgia State Dental Association. - - - - - - 10
Gold Fillings. Method of Consolidating, - - - - - 126
Gold Foils. Soft vs. Adhesive, - - - - - - 380
Gross. Prof. Sam'l D. M.D. - - - - - - - 80
Hare-Lip. Operation for, with Removal of Inter-maxillary Bone. - - - - - - - - - - - - 432
Hare-Lip. Rare form of, - - - - - - - 285
Harriman. G. B. D.D.S. - - - - - - - - 49
Harris. E. N., D.D.S. - - - - - - - - 307
Harvey. L. F., M.D., D.D.S. - - - - - - 506
Headache. Treatment of, - - - - - - - - 328
Heavy Gold Foil. - - - - - - - - - 48
Hemorrhage Following Extraction of a Tooth. - - - 357
Hemorrhoids. A Cure for, - - - - - - - 139
Hydrate of Chloral. - - - - - 24, 43, 90, 225, 234
Hydrate of Chloral. A Test for the Purity of, - - 285
Hydrate of Chloral. Toxical Effects and Fatal Results of, - 4C8

Hydrate of of Chloral. External Application of, . . 570
Hodgkin. J. B., D.D.S. - - - - - - - - 395
Hollace. N. H., M.D. - - - - - - - - - 490
Holmes. Oliver W., M. D. . . . . . . . 542
Hypodermic Injections. Safety of, - - - - - - - 86
Inpaction of Molar Teeth in Left Bronchus. - - - 81
Importance of Right Side of Brain,. - - - - - - - 135
Inhalation of Oxygen on the Pulse. Effect of the, – - 83
Infection. Ready Mode of Preventing. – – – 474
Influence of Diseased Teeth upon the Health. – – – 490
Innocent and Malignant Growths. – – – – 283
Jarvis. O. A., D.D.S. . . . , . . . . . 558
Kansas State Dental Society. – – – – – – 60
Keeping Cavities Dry. - – - – – – 300
Keesee. G. F., D.D.S. – – – - – – - 272
Koch. Dr. C. E. . . . . . . . . 319, 372, 438
Lake Erie Dental Association. . . . . . . . 158
Leblanc. L. J. B., D.D.S. . . . . . . . . 70
Lead Line on the Gums. . . . . . . . . . 235
Lemon for a Cough. . . . . . . . . . . 423
Local Anæsthesia. . . . . . . . . . . 426
Local Application of Carbolic Acid in Diptheria. . . 138
Local and Superficial Anæsthetic Effects of the Phenols. . 137
Life-Like in Death. - - - - - - - - 136
Maggots in the Ear. - - - - - - - - - 89
Malignant Diseases of the Jaws. - - - - - 139
McDonald. R. A., D.D.S. - - - - - - - - 10
McGrau. Dr. T. H. - - - - - - - - 132
McLain. Dr. D. B. - - - - - - - 359, 465, 540
McQuillen. Prof. J. H., M.D., D.D.S. . . . . . 420
McSherry. Jno. M.D. . . . . . . . . . 371
Medical Graduates in the United States. . . . . . 139
Microscopy of the Teeth. . . . . . . . . . 241
Method of Applying Nitrate of Silver and Chloride of Zinc. 288
Methylene as a Anæsthetic. Advantages of, . . . . 331
Minute Structure of an Amalgam Filled Tooth. . . 63
Mitchell's Process for External Tumors . . . . . 181
Mixing Plaster for Impressions. . . . . . 42
Mustard to make Leeches Take. . . . . 85
Naughty Case. . . . . . . . . . 461
Necrosis of Eight Teeth from a Blow. . . . . 179
Necrosis of Lower Jaw from Mercurial Salivation. . . 132
Necrosis of Maxillary Bones Caused by Salivation. . 105
Nervous Force. . . . . . . . . . . 557
Neuralgia. . . . . . . . . 366, 570
Neuralgia. Case of Facial, . . . . . . 371
Neuralgia. Citrate of Caffein in, . . . . . . 331
Neuralgia of Jaw Following Teeth Extraction. . . . 39

New Plastic Material. . . . . . . . . . . . 87
Nitrous Oxide Gas. Condensed Form of, . . . . . 237
Nitrous Oxide. Liquid Form as an Anæsthetic. . . . . 516
Nitrous Oxide. Use (not abuse) of, . . . . . . . 534
No Life Without Phosphorus. . . . . . 236
Nostalgia a Cause of Insanity. . . . . 137
O'Connell. P. A., M.D. . . . . . . . 173
Oil of Peppermint as an Anæsthetic. . . . . 41
Ossified Pulps. . . . . . . . 385
Oxychloride of Zinc for Dental Purposes. . . 130
Oxygen Gas in Pulmonary Diseases. . . . . . 522
Obituary Notices.
Drs. H. C. Peebles. . . . . . . . 144
P. Preterre. . . . . . . . 144
R. G. Snow. . . . . . . . 192
T. J. Crow. . . . . . . . 240
Warren Johnson. . . . . . 240
B. T. Whitney. . . . . . . . . . 576
Painless Extraction of Teeth. . . . . . . . 569
Paralysis Following Dentition. . . . . . 80
Patents. . . . . . . . . . . 342
Patterson. Dr. J. D. . . . . . . . . 60
Pyroxyline Base. A Method of Working, . . . 93
Pyroxyline Dissolved in Oils. . . . . . 331
Permanganate of Potash Injections. . . . . 285
Perry. S. G., D.D.S. . . . . . . . 126
Petroleum. A New way of Testing, . . . . 476
Poisoned Gloves. . . . . . . . . 476
Poisoning by Hydrate of Chloral. . . . . 234
Porter. Dr. J. M. . . . . . . . . 166
Poisoning from Zinc. . . . . . . . 427
Poisoning by Carbolic Acid. Treatment of, . . . . 329
Posture of the Head in Sleeping. . . . . . 426
Present Development and Tendencies of the Dental Profession. 205
Preston. R. H., M.D. . . . . . . . 273
Pulps. Exposed, . . . . . . . . 433
Pus. Formation of, . . . . . . . . 235
Pus. Test Solution for, . . . . . . . 428
Quick Method of Making Dies. . . . . . 30
Red Blood Corpuscles. Structure of, . . . . 466
Replantiug of Teeth. . . . . . . . . 217
Reply to Rubber Company's Chemical Advocate. . 537
Ring Worm. Remedy for, . . . . . . . 135
Robinson. Dr. H. E. . . . . . . . 166
Sage. Dr. H. L. . . . . . . . 279, 499
Salivary Calculus. . . . . . . . 382
Salivation Consequent upon Artificial Teeth. . . . 330
Savage. Dr. R. A. . . . . . . . . 14

Shadoan. W. H., D.D.S. - - - - - 554
Simple Test for Arsenic, Antimony and Phosphorus. - - 422
Smith. Henry, F.R.C.S. - - - - - - 78
Smith. H. A., D.D.S. - - - - - - 130
Smith. A. H., M.D. - - - - - - 168
Smith. T. B., M.D. - - - - - - - 230
Smith. C. Stoddard, D.D.S. - - 264, 289, 308, 343, 400
Smith. N. R., M.D. - - - - - - 408
Smith. T. Curtis, M.D. - - - - - 15
Soda Mint. - - - - - - - 427
Soluble Glass and its Applications. - - - 565
Southern Dental Association. - - - - 189, 193
South Carolina State Dental Association. - - - 61
Spontaneous Combustion of the Human Body. - - 86
Spier. S. F. M.D. - - - - - 116
Spoon Feeding. - - - - - - 282
Splitting a Tooth by Decomposition of the Pulp. - 420
Squibb. E. R., M.D. - - - - - - - 24
Statistics of Dental and Medical Professions. - - - 237
Stevens. A. P., D.D.S. - - - - - - - 143
Styptic Cotton. - - - - - - - - - 573
Sulphur in Croup. - - - - - - - - 88
Sulpho Urea. - - - - - - - - - 428
Sulphate of Nickel in Neuralgia. - - - - - 283
Surgical Diseases of the Mouth. - - - - - - 175
Swallowing Artificial Teeth. - - - - - 46, 78, 94
Teeth at Birth. - - - - - - - - - 93
Temporary Teeth. - - - - - - - 319, 372
Tennessee Dental Association. - - - - - - 287
The Lapse. - - - - - - - - - - 183
The Title of Doctor. - - - - - - - - 89
Thompson. Jas. F., D.D.S. M.D. - - - - - 391
Test for Genuineness of Silver. - - - - - - 427
Test for Arsenic. - - - - - - - - 427
The Dental Cosmos. . . . . . . . 430
The Case Book. . . . . . . . 493
Theory of Nervous Force. - - - - - 557
Tichborne. C. R. C., F.R.C.S. . . . . . 568
Tin Foil. - - - - - - - - - - 134
To Crystallize Plants, Flowers, &c. - - - - - 233
To Polish Marble. - - - - - - - - 42
To Avoid the Ague. - - - - - - - - 424
Torsion. - - - - - - - - - - 527
Treating of Soft Tissues of the Mouth. - - - - - 47
Treatment of Snake Bite. - - - - - - 84
Treatment of Enlarged Tonsils in Children. - - - 86
Transplanting Teeth. - - - - - - - 333
Transplanting Mucous Membrane. - - - - - - 424

Transmission of a Predisposition to Diseases by Inheritance. 230

Troutman. Dr. J. A. - - - - - - 30

Tobacco in Diseases of Nerve Centres. Influence of, - 424

Tripp. Dr. G. W. - - - - - - - 519

Use (not abuse) of Nitrous Oxide. . . . . 514

Use of Microscope in Diagnosis of Morbid Growth. - 116

Uses of Carbolic Acid. . . . . . . 477

Vaccination. The Protective Power of, . , 377

Vaccine Virus. Preserving, , , . . , 334

Varnish. A Useful, . . . . , 376

Vulcanite Plate Retained in the Stomach. . , . 381

Vulcanite Plates; Are they Injurious? , . . 332

Vegetable Carbolic Acid. . . . . . 475

Virginia State Dental Association. . . 272, 287. 395

Walker. J. R., D.D.S. . . . . . 537

Walter. L. D., D.D.S. . . . . , 134

Watson. B. A., M.D. , , . , . 414

Weston's Metal. . . . . . . 142

Wheeler. Dr. E. G. . . . . . , 463

White Gutta Percha. , . . . , 573

White Sulphur Springs Meeting. . . , 59

Wilder. Alex., M.D. . . . . . . , 366

Xylol, the New Remedy for Small Pox. . . 568

Zinc Poisoning. . . . . . . . 427

### ARTICLE IV.

### *Alabama Dental Association.*

The Alabama Dental Association met at Selma, the President, J. G. McAuley, in the chair. The increased attendnce shows a growing interest in the association.

Dr. J. G. McAuley, of Selma, was elected delegate to represent the interests of the Association at the next meeting of the American Dental Association. Dr. Sam'l Rambo and J. W. Keys, of Montgomery, alternates.

Dr. W. J. Reese, of Montgomery, was elected delegate to represent the interests of the same at the next meeting of the Southern Dental Association, at Charleston. Dr. Wm. Deason, of Mobile, alternate.

The following officers were elected for the ensuing year: *President*, Sam'l. Rambo, Montgomery; 1*st Vice President*, Wm. Deason, Mobile; 2*nd Vice President*, J. W. Keys, Montgomery; *Cor. Sec*, W. J. Reese, Montgomery; *Rec. Sec.* and *Treas.*, Robt. A. Savage, Mobile.

The next meeting of the Association will be held in Mobile, on the 16th of August 1871.

Robt. A. Savage,
Sec. Ala. Dental Association.

### TO READERS AND CORRESPONDENTS.

Communications intended for publication, and exchange journals and papers, should be addressed to the EDITOR OF THE AMERICAN JOURNAL OF DENTAL SCIENCE, 259 N. Eutaw St., Baltimore, Md. All letters relating to the business of the journal, or containing cash remittances, to be directed to Messrs. SNOWDEN & COWMAN. Articles for publication should be received by the editor at least three weeks previous to the first day of the month on which the number in which it is designed for them to appear, is issued.

### CONTENTS:

### ORIGINAL COMMUNICATIONS.

### MONTHLY SUMMARY.

Carbolic Acid Preparations .... 40

Oil of Peppermint as an Anæsthetic .... 41

California Quicksilver .... 41

Mixing Plaster Impressions .... 42

Treating Discolored Teeth .... 42

To Polish Marble .... 42

Hydrate of Chloral .... 43

Death from Chloroform .... 43

Bleaching Beeswax .... 44

Death from Inhalation of Ether .... 44

Cancrum Oris .... 44

### EDITORIAL DEPARTMENT.

Dental Items.—Calcified Pulp .... 45

Swallowing Artificial Teeth .... 46

Treating of Soft Tissues of the Mouth .... 47

Heavy Gold Foil .... 48

Alveolar Abscess .... 48

☞ The American Journal of Dental Science will contain fifty pages of reading matter in each number, making a volume of not less than

*Six Hundred* pages;

EXCLUSIVE OF ADVERTISEMENTS.

THE

### AMERICAN JOURNAL

OF

### DENTAL SCIENCE.

**F. J. S. GORGAS, M. D., D. D. S., Editor.**

The *Journal* is issued on the first of each month and contains not less than *fifty* pages of reading matter in each number, exclusive of advertisements, making a volume of *six hundred* pages per annum.

In each number will be found Original Communications, Correspondence, Selected Articles, Monthly Summary, Bibliographical Notices and Editorial Matter, and every effort will be exerted to render the *Journal* worthy of the patronage of the Dental profession.

A generous co-operation is respectfully solicited from the members of the profession; and as the *Journal* has now a printing office of its own which materially lessens the cost of its publication, it will be issued at the reduced subscription price of

**$2.50 Per Annum in Advance.**

Communications are respectfully solicited on all subjects pertaining to the practice of dentistry, as well as reports of cases occurring in dental practice.

### RATES OF ADVERTISING.

| | 1 MO. | 2 MO. | 3 MO. | 6 MO. | 12 MO. |
|---|---|---|---|---|---|
| 1 PAGE. | $15 00 | $22 50 | $30 00 | $50 00 | $100 00 |
| ½ " | 10 00 | 15 00 | 20 00 | 30 00 | 50 00 |
| ¼ " | 7 00 | 11 00 | 15 00 | 20 00 | 30 00 |
| ⅛ " | 5 00 | 8 00 | 11 00 | 15 00 | 20 00 |

All original communications designed for publication, works for review, new instruments for notice, exchanges, &c., should be directed to the editor, 259 N. Eutaw St., Baltimore, Md.

All advertisements and subscriptions should be addressed to

SNOWDEN & COWMAN,
*Publishers of the Am. Journal of Dental Science.*
86 W. FAYETTE ST. BET. CHARLES & LIBERTY STS.,
BALTIMORE, Md

